# The mediating role of academic satisfaction and examination-related stress in explaining the association between academic grit and university students’ subjective well-being

**DOI:** 10.3389/fpsyg.2026.1884240

**Published:** 2026-07-02

**Authors:** Ernesto Lodi, Lucrezia Perrella, Rita Zarbo, Giovanni Maria Natale, Patrizia Patrizi

**Affiliations:** 1 Department of Humanities and Social Sciences, University of Sassari, Sassari, Italy; 2 Department of Human and Social Sciences, University of Enna Kore, Enna, Italy

**Keywords:** academic grit, academic satisfaction, academic stress, subjective well-being, university students

## Abstract

**Introduction:**

Subjective well-being is increasingly recognized as crucial for the psychological adjustment and academic functioning of university students. Academic grit may represent an important resource for students’ well-being, but the mechanisms underlying this association remain insufficiently understood. This study aimed to examine the role of academic satisfaction and examination-related stress in mediating the relationship between academic grit and subjective well-being.

**Methods:**

A convenience sample of 778 university students (age range: 19–69, M-age = 27.9, SD-age = 9.9) participated in the study. The study included the following instruments: Academic Grit Scale, College Satisfaction Scale (C-Sat), Examination stress sub-scale of E-CEA, Well-being Profile (WB-Pro). Data were analysed using Structural Equation Modeling (SEM) combining latent and observed variables and maximum likelihood estimation in LISREL.

**Results:**

Academic grit was directly and indirectly associated with subjective well-being through academic satisfaction and examination-related stress. Academic satisfaction emerged as the primary indirect pathway linking academic grit to subjective well-being, whereas examination-related stress played a smaller, although still significant, role. The overall model explained 48.5% of the variance in subjective well-being (*R*^2^ = 0.485).

**Conclusion:**

Academic grit may represent a relevant psychosocial resource for university students’ subjective well-being. Its association with well-being appears to be partly explained by higher academic satisfaction and lower examination-related stress. These findings suggest that university well-being interventions should consider both the promotion of positive academic experiences, and the management of examination demands.

## 1 Introduction

Student well-being in the university setting is now widely recognised as a crucial factor in psychological adjustment and academic success. Nowadays, university students are increasingly exposed to complex academic demands (Li, 2025; Zhao et al., 2025), pressures related to performance and psychological vulnerability (Barbayannis et al., 2022; Fang et al., 2025), as well as greater uncertainty about the future (Ryan and Baik, 2026). These factors can negatively impact their well-being, which is why it is essential to understand those that promote or hinder it.

Subjective well-being has become a highly relevant issue in higher education, as the literature indicates that many university students struggle to maintain a positive perspective on their lives and academic experiences. For example, recent studies have highlighted a significant decline in subjective well-being among university students (Baetens et al., 2022; Donald and Jackson, 2022), and lower levels of subjective well-being are associated with greater academic adaptation difficulties, lower academic satisfaction and increased vulnerability to psychological distress (Franzen et al., 2021; Zalazar-Jaime et al., 2022). Conversely, higher levels of subjective well-being have been associated with more adaptive functioning (Marsh et al., 2020), better academic performance (Britwum, 2025) and greater perseverance in university studies (Baik et al., 2019). These findings highlight the importance of identifying the personal and contextual factors that promote subjective well-being, to help the development of interventions aimed at supporting students’ success and perseverance during their university studies. University is a living community where students also develop in their social roles. Looking after their wellbeing should be a goal pursued by the entire academic community. Moreover, these aspects are evident to us too through our experience of running the university counselling service, where the university is not only a place of academic achievement but also of individual and social growth. It is precisely for these reasons that we have launched a project involving experimental training to improve subjective well-being according to the dimensions of the Well-being Profile scale, such as the PRIN project “Paths towards well-being: An action-research project to promote quality of life from a multidimensional perspective.”

Historically, studies of well-being have often viewed it in utopian terms and focused on objective measures such as happiness and life satisfaction. Recent scientific literature, however, has highlighted that well-being is a multidimensional construct and that understanding it therefore requires integrating the eudaimonic perspective (Marsh et al., 2020; Perrella et al., 2026a,b). Indeed, we can define well-being as a construct emerging from the personal, emotional, and social domains (Keyes, 2002; Marsh et al., 2020). Consistent with this comprehensive approach, the Well-Being Profile (WB-Pro) is adopted in this study as a multidimensional framework for operationalising well-being (Marsh et al., 2020; Scalas et al., 2023).

Among the psychosocial resources that can promote students’ subjective and domain-specific well-being, academic grit, defined as each student’s perseverance in pursuing their academic goals, plays an important role (Clark and Malecki, 2019). Academic grit is associated with greater perseverance and persistence (Harpaz et al., 2024), motivation (Wu et al., 2022), engagement (Harpaz et al., 2024), and resilience (Weng and Peng, 2026) towards academic challenges, as well as lower academic drop-out rates (Woodward et al., 2026), characteristics associated with more positive psychological outcomes, including greater well-being (Tang and Zhu, 2024). However, although the association between academic grit and well-being is widely explored in the literature, the psychosocial factors through which this association may operate remain underexplored.

Two potential psychosocial factors that could explain this association are academic satisfaction and exam stress. Academic satisfaction represents students’ cognitive and affective evaluation of their academic experience (Lodi et al., 2017) and is associated with higher levels of subjective well-being (Zalazar-Jaime et al., 2022); it is also a central element in adapting to university life (Emaliana et al., 2025; Perrella et al., 2026a,b). Several studies (e.g., Datu, 2021; Harpaz et al., 2024; Khoirunnisa et al., 2023) highlight that resilient students may be more likely to perceive their efforts as meaningful and rewarding, to maintain commitment to academic goals, to cope with academic challenges, and to persevere despite difficulties (Bernardo et al., 2025; Ibrahim et al., 2025). This, in turn, may increase their domain-specific satisfaction and contribute to greater overall well-being (Lent et al., 2007; Lent and Brown, 2008; Zalazar-Jaime et al., 2022). Therefore, academic satisfaction may represent a significant resource in the relationship between academic grit and subjective well-being, as academic grit could promote more positive evaluations of the academic experience.

At the same time, academic contexts are characterised by multiple stressors (Cabanach et al., 2016; Reddy et al., 2018). Among these, examination-related stress represents a major risk factor for a student’s well-being (Ansari et al., 2025; Dick et al., 2025; Reddy et al., 2018) and is linked to increased psychological distress (Dick et al., 2025; Tran et al., 2022), particularly increased perceived anxiety (Koudela-Hamila et al., 2022) and burnout (Barbayannis et al., 2022), which in turn may lead to university dropout (Barbayannis et al., 2022; Blanco et al., 2025). Students with high academic grit may be able to cope more effectively with the challenges associated with university exams and perceive them as more manageable (Kannangara et al., 2018; Omar et al., 2026). Consequently, this could help raise their levels of subjective well-being, as academic grit may reduce the perceived burden of university examination-related demands and increase productive efforts towards to achieve academic goals.

Although previous studies have reported positive associations between academic grit and subjective well-being, there is still limited knowledge regarding the mechanisms through which this relationship emerges in higher education settings. Research has rarely examined the contribution of resources such as academic satisfaction and specific academic stressors, such as examination-related stress, in explaining the association between academic grit and subjective well-being. Furthermore, whilst several studies have focused on general indicators of academic stress, the role of examination-related stress as a potential explanatory mechanism remains under-explored. Therefore, the present study aims to examine the association between academic grit and subjective well-being and to test whether this relationship is mediated by academic satisfaction and exam stress. Specifically, the hypothesis is that academic grit has both direct and indirect effects on subjective well-being, mediated by academic satisfaction and examination-related stress. Although the cross-sectional design of the study does not allow for causal inferences, the proposed model is based on existing theoretical and empirical evidence and may provide a useful framework for better understanding the associations between the variables under investigation.

## 2 Literature review

### 2.1 Academic satisfaction

Academic satisfaction reflects students’ positive evaluation of their role and experiences within the university context (Lent et al., 2007). It concerns both current experiences and future expectations regarding the achievement of academic goals (Kumar and Dileep, 2006) and influences career choices (Zarbo et al., 2026) and the general well-being of university students (Ma and Mustapha, 2025). Academic satisfaction has also been shown to be positively associated with several dimensions of students’ subjective well-being, as conceptualized by the WB-Pro 15 model adopted in the present study, including autonomy, perceived competence, and engagement (Froment et al., 2023; Gutiérrez and Tomás, 2018; Levpušček and Podlesek, 2019).

Academic satisfaction has emerged as a strong correlate of students’ mental health and psychological functioning, alongside traditional indicators such as stress (Zarbo et al., 2025). In fact, it is associated with greater psychological well-being, less distress, and better adaptation to university life (Franzen et al., 2021; Tran et al., 2022), whilst also playing a mediating role in the relationship between perceived stress and well-being, mitigating the impact of difficulties on students (Qin et al., 2025).

Empirical findings also suggest that students with higher levels of academic satisfaction report greater life satisfaction, more positive expectations for the future, a sense of fulfillment from their studies, and a greater sense of meaning and purpose in their studies (Carranza Esteban et al., 2022; Jadrić et al., 2025; Lodi et al., 2021; Zalazar-Jaime et al., 2022). These patterns extend to motivational and behavioural outcomes, including greater perseverance in effort, a key component of academic grit and an important predictor of educational success (Bowman et al., 2015), as well as a higher likelihood of completing a degree program (Gopalan and Brady, 2020) and a reduced risk of dropping out (Gonzalez et al., 2025).

Finally, academic satisfaction has been linked to lower levels of stress and reduced risk of burnout (Bagdžiūnienė et al., 2025; Qin et al., 2025), highlighting its central role in both students’ academic trajectories and psychological adaptation.

Overall, these findings seem to suggest that academic satisfaction may act as a potential mediating mechanism between academic grit and subjective well-being. Students with higher levels of academic grit might be more inclined to persist in pursuing their academic goals, to perceive their efforts as meaningful, and to evaluate their academic experiences more positively. Given the well-established link between academic satisfaction and subjective well-being, academic satisfaction may contribute to explain how academic grit is associated with students’ subjective well-being.

### 2.2 Examination-related stress

Examination-related stress is a distinct domain-specific stressor within the academic environment within the broader academic stress framework (Cabanach et al., 2016; Perrella et al., 2024). It can be conceptualized as a domain-specific form of academic stress that emerges in evaluative contexts, particularly during exam preparation and performance. According to the transactional model of stress proposed by Lazarus and Folkman (1984), stress is a product of the interaction between environmental demands and the individual’s cognitive appraisal of those demands in relation to their perceived coping resources. In academic contexts, this process is shaped by specific stressors, including examinations, workload, interpersonal dynamics, and organizational factors (Cabanach et al., 2016; Reddy et al., 2018; Verma et al., 2002). Among these stressors, examinations represent a recurrent and particularly demanding context, involving workload demands, evaluation pressure, fear of failure, and potential negative consequences for academic progression (Cabanach et al., 2010; Reddy et al., 2018). Thus, examination-related stress can be better understood in terms of the perceived stressfulness of examination-related demands, rather than as a purely emotional response. This distinction is consistent with appraisal-based models of stress and helps differentiate examination-related stress from related constructs such as test anxiety. Empirical evidence indicates that examination-related stress is associated with poorer well-being, lower academic satisfaction, and increased psychological vulnerability, including symptoms of anxiety and depression (Ang and Huan, 2006; Deb et al., 2015). However, these associations are not uniform, as individual resources such as self-efficacy, coping strategies, and emotional regulation can mitigate the impact of examination-related demands (Karaman et al., 2019; Kurtović et al., 2018). Developmental differences in cognitive and emotional dimensions may also influence how individuals manage stressful situations (Guarnera et al., 2024). Despite the centrality of examinations in students’ academic experience, two main limitations emerge in the literature. First, research has predominantly focused on test anxiety, while comparatively less attention has been devoted to examination-related stress. Second, many existing instruments tend to assess general stress or emotional responses rather than specific sources of stress within the academic environment (Bedewy and Gabriel, 2015). In response to these limitations, the present study adopts the examination stress subscale of the Academic Stressors Scale (E-CEA), which conceptualizes examination-related stress as a distinct academic stressor and provides a more specific framework for investigating students’ responses to evaluative demands (Cabanach et al., 2016; Perrella et al., 2024).

Although there are few studies that have analysed exam stress as a mediating variable between academic grit and subjective well-being, the evidence suggests a possible indirect link between the two. Students with higher levels of academic grit tend to report greater perseverance, resilience, and adaptive coping resources, characteristics that may help them perceive examination-related demands as more manageable. Furthermore, lower levels of exam-related stress are associated with better well-being outcomes, including higher levels of subjective well-being. Therefore, exam-related stress may represent a possible mechanism through which academic grit is associated with subjective well-being.

### 2.3 Academic grit

Academic grit refers to students’ ability to maintain effort and commitment toward long-term educational goals despite challenges and setbacks. Originally defined by Duckworth et al. (2007), the construct of grit comprises two dimensions: consistency of interests and perseverance of effort. Applied to educational settings, this construct has been identified as a relevant factor in students’ academic adjustment and well-being (Tang and Zhu, 2024). Consistent with this perspective, several studies have identified grit as a significant predictor of academic achievement and persistence (Lam and Zhou, 2022; Sulla et al., 2018; Wang, 2021). Beyond academic performance, grit has also been associated with several adaptive psychological and behavioral outcomes across educational levels, including motivation, student engagement, psychological resilience, passion, social competence, and overall life satisfaction (Liao and Chen, 2022; Schimschal et al., 2022; Tang et al., 2023). Academic grit also appears to function as a protective factor against maladaptive behaviors that may interfere with effective learning (Wolters and Hussain, 2015). More broadly, previous research suggests that grit may buffer the negative impact of stressful and adverse educational experiences, while being associated with lower levels of anxiety, depression, and general psychological distress (Kalia, 2021; Zhang et al., 2018).

In this sense, grit can be understood as an important resource that supports students’ psychological well-being and contributes to higher levels of happiness and life satisfaction (Vainio and Daukantaitė, 2016). Recent evidence has also linked academic grit to executive functions (Liao and Chen, 2022) and emotional regulation (Abdelshafy et al., 2025), suggesting its relevance for both cognitive and affective functioning. Moreover, emotional regulation abilities associated with academic grit may help students manage academic stress more effectively (Abdelshafy et al., 2025). These findings suggest that academic grit functions as an adaptive psychological resource that help students cope with academic challenges while promoting persistence, adjustment, and psychological well-being in educational settings (Tang and Zhu, 2024).

Taken together, these findings suggest that academic grit is an adaptive psychological resource that can support students’ perseverance, adjustment and subjective well-being. However, although previous studies have documented the positive association between academic grit and subjective well-being, the mechanisms underlying this relationship remain largely unexplored. Academic satisfaction and exam-related stress may represent two mechanisms that could help explain the association between academic grit and subjective well-being, reflecting both a positive evaluation of the academic experience and the ability to effectively manage exam-related demands.

### 2.4 Subjective well-being

Within psychological research, subjective well-being is commonly understood as individuals’ evaluation of their own lives, encompassing both cognitive judgments and affective experiences (Diener, 1984). Building on this multidimensional perspective, the Well-Being Profile (WB-Pro) model, adopted in the present study, reflects an integrated approach that extends beyond the traditional distinction between hedonic and eudaimonic well-being (Marsh et al., 2020; Scalas et al., 2023). Within this model, well-being is not reduced to the absence of psychological distress, but is instead defined as a constellation of psychological, relational, and social resources indicative of optimal functioning. The WB-Pro operationalises this construct through 15 interrelated dimensions: autonomy, clear thinking, competence, emotional stability, empathy, engagement, meaning, optimism, positive emotions, positive relationships, prosocial behavior, resilience, self-acceptance, self-esteem, and vitality (Marsh et al., 2020).

Consistent with this broad conceptualization, subjective well-being has been associated with adaptive functioning, improved quality of life, and greater life satisfaction (Buecker et al., 2023; Diener and Chan, 2011; Diener et al., 2017). In educational settings, these associations extend to students’ adjustment, satisfaction, and performance. Subjective well-being has been shown to relate positively to academic satisfaction (Zhou et al., 2025) and to academic performance and outcomes (Zalazar-Jaime et al., 2022; Chattu et al., 2020). Furthermore, it is associated with the development of adaptive academic competences, such as time management and study organization, which may help students manage educational demands effectively and support their academic success (Nonis and Hudson, 2010).

In addition to performance-related outcomes, subjective well-being is linked to more positive motivational and behavioural patterns, including higher levels of academic engagement, persistence, and commitment to university studies (Liu et al., 2024; Passeggia et al., 2023; Puiu et al., 2024). It is also positively associated with psychological and interpersonal resources, such as resilience (Tam et al., 2020), autonomy (Wang, 2021), adaptability, and social connectedness (Feraco et al., 2023). Subjective well-being may also play a protective role in students’ mental health, as higher levels of well-being are associated with lower perceived stress and a reduced likelihood of anxiety and depressive symptoms (Chattu et al., 2020; Liu et al., 2024; Zalazar-Jaime et al., 2022).

Overall, the literature suggests that subjective well-being is closely linked to both students’ academic experiences and their psychosocial resources. A positive assessment of the university experience (i.e., academic satisfaction) and reduced levels of stress are important factors associated with well-being. At the same time, resources such as academic grit may enable students to persevere in their studies despite difficulties. Examining these relationships together could therefore provide a greater understanding of the factors associated with subjective well-being in the university context.

## 3 Aims of the study and hypothesis

Based on the theoretical framework, this study examined the mediating roles of academic satisfaction and examination-related stress in the relationship between academic grit and subjective well-being in a college student sample. The hypotheses of this study are as follows:

Hypothesis 1 (H1): Academic grit is positively associated with subjective well-being.

Hypothesis 2 (H2): Academic satisfaction mediates the relationships between academic grit and subjective well-being.

Hypothesis 3 (H3): Examination-related stress mediates the relationships between academic grit and subjective well-being.

## 4 Methods and procedures

### 4.1 Participants

A total of 778 university students took part in the survey. Participants were aged 19–69 (M = 27.9; SD = 9.9). 71.7% were women and 28.3% were men. The sample was predominantly Italian (97.2%). 71.7% of participants were attending a three-year undergraduate degree program, 9.9% a master’s degree program, 12.6% a single-cycle master’s degree program and 5.8% a postgraduate program. 18.6% are studying medical and health sciences, 65% humanities and social sciences, and 16.3% economic and law-related disciplines.

### 4.2 Instrument

*Socio-demographic data*: age, gender, nationality, field of study and course attended.

*Academic Grit Scale* (AGS; Clark and Malecki, 2019) in the Italian validation by Sulla et al. (2018). The AGS comprises 10 items rated on a 5-point Likert scale (1 = not at all like me, 5 = very much like me). In this study, both Cronbach’s alpha and McDonald’s Omega coefficient for the whole scale are 0.94.

*College Satisfaction Scale* (C-Sat; Lodi et al., 2017), a scale consisting of 20 items on a 5-point Likert scale (from 1 = not at all to 5 = completely), which assesses satisfaction with the academic experience through 5 sub-dimensions: satisfaction with the choice of course of study, satisfaction with services, satisfaction with relationships, satisfaction with study, and satisfaction with the usefulness of the course of study in relation to future career. In this study, both Cronbach’s alpha and McDonald’s Omega coefficient for the whole scale are 0.92.

*Examination stress*, a sub-scale of the E-CEA scale (Cabanach et al., 2016; Perrella et al., 2024). The scale examines, through six factors, the possible situations and/or events that cause stress in the academic environment. The ‘examination stress’ sub-scale consists of three items on a 5-point Likert scale (1 = never; 5 = always). In this study, the internal consistency of the sub-scale was good, with both Cronbach’s alpha and McDonald’s Omega equal to 0.84, which is considered satisfactory given the small number of items (Nunnally and Bernstein, 1994).

*Well-being Profile* (WB-Pro; Marsh et al., 2020), reduced 15-item Italian version by Scalas et al. (2023). The scale assesses subjective well-being across 15 dimensions: Autonomy, Clear thinking, Competence, Emotional stability, Empathy, Engagement, Meaning, Optimism, Positive emotions, Positive relations, Prosocial behavior, Resilience, Self-acceptance, Self-esteem, Vitality. The scale consists of one item per domain, rated on a 9-point Likert scale (1 = strongly disagree, 9 = strongly agree). In this study, both Cronbach’s alpha and McDonald’s Omega coefficient for the whole scale are 0.90.

### 4.3 Procedure

Before completing the questionnaire, all participants were provided with detailed information about the research objectives and, after reading and signing the informed consent form, completed the online survey. No incentives were given for participation. Data collection took place between March and May 2025 using the Google Forms platform. Participation was entirely voluntary and anonymous. To ensure anonymity and prevent duplicate entries, participants were asked to assign themselves an alphanumeric code consisting of the initials of their first and last names and their date of birth. Participants were recruited primarily on the criteria of convenience, specifically: (a) in university classrooms during lectures; (b) via advertisements on university websites; (c) by sending the questionnaire by email. Furthermore, no exclusion criteria were applied. The questionnaire took approximately 25 min to complete. The study was conducted in accordance with the ethical principles of the Italian Psychological Association, the current version of the Declaration of Helsinki, and the Code of Ethics of the National Order of Italian Psychologists. It was approved by the Ethics Committee of the University of Sassari (approval date 06/12/2022, project no. 2022-UNSSCLE-0061755).

### 4.4 Data analysis

Data analysis was conducted in two phases using IBM SPSS Statistics (version 30.0) and LISREL (version 8.80). First, SPSS Statistics was used to calculate descriptive statistics and correlations with Pearson coefficient and to conduct a preliminary analysis about: (a) multicollinearity, assessed using variance inflation factors (VIF) and tolerance values (Hair et al., 2019); (b) the presence of missing values; (c) sociodemographic differences in relation to the variables under study. Second, the hypothesized mediation model was tested using Structural Equation Modeling (SEM) with latent and observed variables and maximum likelihood estimation in LISREL. Model fit was evaluated using multiple fit indices such as the chi-square statistic (χ^2^), the chi-square/degrees of freedom ratio (χ^2^/df), RMSEA, SRMR, CFI and NNFI. The significance of the indirect associations was assessed using Monte Carlo simulation procedures with confidence intervals based on 20,000 replications. Given gender imbalance (71.7% women), a gender-based post-stratification weighting procedure was applied to improve the sample’s representativeness. The demographic reference parameters were derived from statistical data on the gender distribution of students at the University of Sassari. The weights were calculated using the ratio of students’ population proportions to sample proportions and applied as part of a sensitivity analysis.

## 5 Results

### 5.1 Preliminary analyses

Descriptive statistics and bivariate correlations among the study variables are presented in Table 1. All variables were significantly correlated in the expected directions. Academic grit showed a moderate positive association with subjective well-being (*r* = 0.558, *p* < 0.01) and academic satisfaction (*r* = 0.581, *p* < 0.01), and negative correlation with examination-related stress (*r* = −0.212, *p* < 0.01). Examination-related stress was negatively associated with both academic satisfaction (*r* = −0.222, *p* < 0.01) and subjective well-being (*r* = −0.377, *p* < 0.01). Academic satisfaction was positively associated with subjective well-being (*r* = 0.542, *p* < 0.01).

**Table 1:** Descriptive statistics and bivariate correlations among study variables.

Variables	M	SD	1	2	3	4
1. AGS	37.01	20.03	—			
2. Examination stress	9.49	3.26	−0.212**	—		
3. C-Sat	72.23	13.88	0.581**	−0.222**	—	
4. WB-Pro	97.36	20.04	0.558**	−0.377**	0.542**	—

*N* = 778. *p < 0.05, **p < 0.01.

Preliminary analyses did not reveal any missing values or sociodemographic differences in relation to the variables under study. The results remained unchanged after post-stratification weighting, indicating that the observed associations were not influenced by the sample’s gender composition. Prior to model estimation, multicollinearity was examined and no concerns emerged, as all VIF values were low (range = 1.02–1.56) and tolerance values were within recommended thresholds (range = 0.64–0.98; Hair et al., 2019). These findings indicated adequate independence among the study variables and supported the estimation of the structural equation model.

### 5.2 Mediation model

The model seems demonstrate a good/acceptable fit to the data, χ^2^(1074) = 8097.06, χ^2^/df = 7.54, RMSEA = 0.10, SRMR = 0.08, CFI = 0.93, and NNFI = 0.93. Academic grit explained 35.1% of the variance in academic satisfaction (*R*^2^ = 0.351) and 4.9% of the variance in examination-related stress (*R*^2^ = 0.049). The full model explained 48.5% of the variance in subjective well-being (*R*^2^ = 0.485).

**Figure 1:**
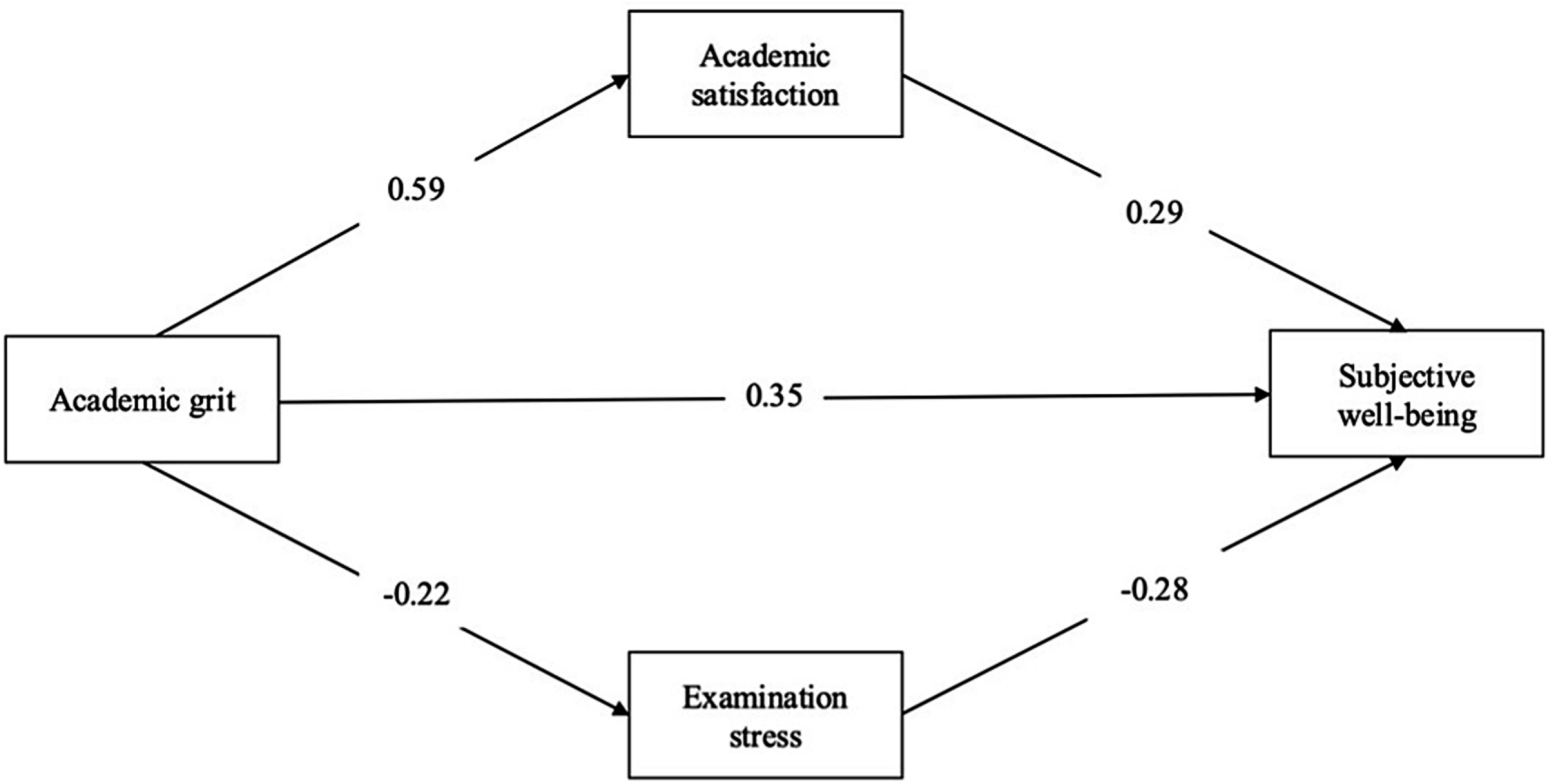
Structural equation model of the associations among academic grit, academic satisfaction, examination stress, and subjective well-being.

As shown in Figure 1 and Table 2, academic grit was positively associated with academic satisfaction (*γ* = 0.592, *t* = 15.55, *p* < 0.001) and subjective well-being (*γ* = 0.351, *t* = 8.78, *p* < 0.001), and negatively associated with examination-related stress (*γ* = −0.222, *t* = −5.55, *p* < 0.001). Academic satisfaction was positively associated with subjective well-being (*β* = 0.290, *t* = 7.33, *p* < 0.001), whereas examination-related stress was negatively associated with subjective well-being (*β* = −0.280, *t* = −8.12, *p* < 0.001).

**Table 2:** Structural model results.

Paths	Coefficient	*t*	*p*
AGS ⇒ C-Sat	γ = 0.592	15.55	< 0.001
AGS ⇒ Examination stress	γ = −0.222	−5.55	< 0.001
AGS ⇒ WB-Pro	*γ* = 0.351	8.78	< 0.001
C-Sat ⇒ WB-Pro	β = 0.290	7.33	< 0.001
Examination stress ⇒ WB-Pro	β = −0.280	−8.12	< 0.001

γ coefficients represent paths from exogenous to endogenous variables; β coefficients represent paths among endogenous variables.

### 5.3 Indirect effects

As shown in Table 3, the indirect association between academic grit and subjective well-being through academic satisfaction was significant (indirect effect = 0.291, 95% CI [0.207, 0.379]). Similarly, the indirect association through examination-related stress was also significant (indirect effect = 0.104, 95% CI [0.063, 0.152]). Since neither confidence interval included zero, both indirect associations were considered statistically significant.

**Table 3:** Indirect associations.

Paths	Indirect effect	95% CI
AGS ⇒ C-Sat ⇒ WB-Pro	0.291	[0.207, 0.379]
AGS ⇒ Examination stress ⇒ WB-Pro	0.104	[0.063, 0.152]

Confidence intervals based on Monte Carlo simulation with 20,000 replications.

## 6 Discussion

The present study examined the relationship between academic grit and subjective well-being, focusing on the mediating role of academic satisfaction and examination-related stress. The findings support the hypothesized model, indicating that academic grit was positively associated with subjective well-being both directly and indirectly through the proposed mediators. The results should be interpreted considering the model’s fit indices, which are good for some indices and acceptable for others. Indeed, the RMSEA and the χ^2^/df ratio are considered mediocre by conventional standards (Browne and Cudeck, 1993; Hu and Bentler, 1999). One possible explanation may be related to the measurement characteristics of the WB-Pro. The 15-item version assesses the 15 dimensions of well-being using a single item for each domain, thereby reflecting the multidimensional nature of subjective well-being. Consequently, modelling the construct as a single latent factor may have introduced some degree of model misspecification and contributed to the less-than-optimal fit indices. Finally, the RMSEA, which is sensitive to sample size and an increase in degrees of freedom, should also be interpreted considering the quality of the other indices, suggesting a potentially acceptable fit to the data.

About results, academic satisfaction emerged as the strongest indirect pathway, whereas examination-related stress played a smaller but significant role. The Monte Carlo confidence intervals further supported the significance of both indirect associations. Overall, these findings suggest that students with higher levels of academic grit tend to report more positive evaluations of their academic experience and lower levels of examination-related stress, both of which are associated with greater subjective well-being. Furthermore, the results of the SEM analysis confirmed the hypothesised pattern of associations, providing evidence that both academic satisfaction and examination-related stress represent relevant pathways through which academic grit is associated with students’ subjective well-being.

These findings provide further support for conceptualising of academic grit as a psychological resource that operates not only at a dispositional level but also through proximal cognitive and affective mechanisms. In particular, the results suggest that the relationship between academic grit and subjective well-being is not only direct but appears to be closely related to students’ perceptions and experiences of their university environment. A key contribution of the present study concerns the central role of academic satisfaction as a mediator. The findings indicate that academic satisfaction represents the primary mechanism through which academic grit is associated with subjective well-being, highlighting the importance of students’ cognitive and affective evaluations of their academic experience. This interpretation aligns with Social Cognitive Career Theory, which holds that satisfaction reflects the interaction among personal resources, goal pursuit, and contextual factors (Lent et al., 2007; Lent and Brown, 2008). Moreover, prior research has shown that academic satisfaction is strongly associated with well-being and adaptive functioning (Franzen et al., 2021; Zalazar-Jaime et al., 2022).

Examination-related stress also showed a significant, although weaker, mediating effect. Higher levels of academic grit were associated with lower examination-related stress, which, in turn, was related to higher subjective well-being. This finding is consistent with transactional models of stress, which conceptualize stress as a function of the interaction between environmental demands and perceived coping resources (Lazarus and Folkman, 1984). In academic contexts, examination-related demands represent a major source of stress and are associated with negative psychological outcomes (Deb et al., 2015; Reddy et al., 2018).

Importantly, the weaker mediating role of examination-related stress compared to academic satisfaction suggests that cognitive and evaluative processes may play a more central role than stress-related responses in explaining how academic grit is associated with subjective well-being. Academic satisfaction appears to reflect a more stable and global appraisal of the academic experience, whereas examination-related stress captures more situational and context-dependent responses. This distinction highlights the need to further differentiate among types of academic stress, as multidimensional approaches conceptualise it as a set of domain-specific stressors. Furthermore, academic grit explained a substantially larger portion of the variance in academic satisfaction than in examination-related stress, providing additional support for the greater relevance of satisfaction-related mechanisms in understanding students’ subjective well-being.

Overall, the findings support a multidimensional view of subjective well-being as the result of interactions between personal and contextual factors (Marsh et al., 2020). In this sense, academic grit appears to be associated with well-being not only as a dispositional trait, but also through intermediate processes associated with students’ academic experiences.

These results are consistent with previous literature identifying academic grit as a relevant psychological resource associated with adaptive outcomes such as persistence, resilience, and life satisfaction (Schimschal et al., 2022; Tang and Zhu, 2024). In this perspective, students with higher levels of grit may experience greater well-being possibly because of their sustained commitment to long-term goals and their ability to cope with academic challenges (Kalia, 2021; Zhang et al., 2018).

From a practical perspective, the findings suggest that interventions to promote students’ subjective well-being should not focus solely on reducing stress. Given the protective role of academic grit, interventions designed to foster perseverance and the ability to set long-term goals may contribute to more positive evaluation of university students’ academic experience in terms of satisfaction. They may also to be more effective strategies for achieving positive well-being and coping with academic difficulties.

## 7 Limitations and future implications

Despite the strengths of this study, the research has certain limitations. Firstly, a cross-sectional study design was used. Even though a mediation analysis was conducted, the cross-sectional mediation models do not allow us to confirm the causal hypotheses about the relationships among the research variables. This means that the indirect effects assessed in this study are conditional associations rather than causal relationships. For this reason, future research could make use of longitudinal studies or experimental design to establish the temporal order of the variables and verify causal hypotheses with greater rigour. A further limitation concerns the fit of the SEM. Although the model reproduced the hypothesized pattern of associations and all structural paths were statistically significant, some fit indices did not fully meet conventional criteria for good model fit, particularly the RMSEA value.

Self-report measures have significant limitations due to participants’ subjectivity and social desirability biases. Furthermore, they may also have introduced a common-method bias. Future research could consider a multi-methodological approach to improve the validity of the constructs being measured. In addition, a convenience sample may have introduced selection bias and limited the generalisability of the results. Although subgroup analyses were conducted to examine possible differences between socio-demographic variables and study variables, post-stratification weighting based on gender was applied, and sensitivity analyses were carried out to assess the robustness of the results, these procedures cannot fully correct for potential selection bias. Future research should consider these aspects and employ more representative sampling strategies.

## 8 Conclusion

This study contributes to the literature on university students’ well-being by highlighting the protective role of academic grit as a psychological resource associated with subjective well-being. The results suggest that academic satisfaction and, to a lesser extent, examination-related stress may help explain the association between academic grit and subjective well-being. It is therefore essential to consider both cognitive-evaluative mechanisms and stress-related mechanisms when investigating student well-being in higher education settings. The predominant role of academic satisfaction suggests that the well-being of university students is not linked only to stress reduction, but also to the opportunity to have positive and meaningful academic experiences. Overall, these findings may help highlight the importance of promoting academic environments that support university students’ psychosocial resources. These resources could help students cope with the demands and pressures of university life, particularly those related to exams.

## Data Availability

The raw data supporting the conclusions of this article will be made available by the authors, without undue reservation.
